# Steam Explosion (STEX) of *Citrus* × *Poncirus* Hybrids with Exceptional Tolerance to *Candidatus* Liberibacter Asiaticus (CLas) as Useful Sources of Volatiles and Other Commercial Products

**DOI:** 10.3390/biology10121285

**Published:** 2021-12-07

**Authors:** Christina Dorado, Kim D. Bowman, Randall G. Cameron, John A. Manthey, Jinhe Bai, Kyle L. Ferguson

**Affiliations:** U.S. Horticultural Research Laboratory, United States Department of Agriculture, Agricultural Research Service, Fort Pierce, FL 34945, USA; kim.bowman@usda.gov (K.D.B.); randall.cameron@usda.gov (R.G.C.); john.manthey@usda.gov (J.A.M.); jinhe.bai@usda.gov (J.B.); k.ferge@gmail.com (K.L.F.)

**Keywords:** flavonoid, pectic hydrocolloid, peel oil, sugar, valorization

## Abstract

**Simple Summary:**

Orange and grapefruit production in Florida has dropped 75% because of a disease known as Huanglongbing (HLB) which is caused by a bacteria (CLas). Infected trees produce immature, green fruit and eventually die. Methods to treat CLas are very expensive and there is no cure. Many of the valuable products found in oranges and grapefruits can be found in citrus hybrid varieties and these hybrid varieties are known to be naturally very tolerant to CLas. In this work, we used steam treatment followed by water washing for the recovery of valuable citrus products from three citrus hybrids, US-802, US-897, and US-942. For most of the products, more than 80% could be recovered from the hybrids using steam treatment followed by water washing. These citrus hybrids, therefore, have the potential to be an alternative citrus crop that thrives in an HLB environment producing economically valuable products that are recovered in high yields in an environmentally friendly way.

**Abstract:**

Florida citrus production has declined 75% due to Huanglongbing (HLB), a disease caused by the pathogenic bacterium *Candidatus* Liberibacter asiaticus (CLas). Methods to combat CLas are costly and only partially effective. The cross-compatible species *Poncirus trifoliata* and some of its hybrids are known to be highly tolerant to CLas, and thus can potentially serve as an alternative feedstock for many citrus products. To further investigate the commercial potential of citrus hybrids, three citrus hybrids, US-802, US-897, and US-942, were studied for their potential as feedstocks for citrus co-products using steam explosion (STEX) followed by water extraction. Up to 93% of sugars were recovered. US-897 and US-942 have similar volatile profiles to that of the commercial citrus fruit types and as much as 85% of these volatiles could be recovered. Approximately 80% of the pectic hydrocolloids present in all three hybrids could be obtained in water washes of STEX material. Of the phenolics identified, the flavanone glycosides, i.e., naringin, neohesperidin, and poncirin were the most abundant quantitatively in these hybrids. The ability to extract a large percentage of these compounds, along with their inherent values, make US-802, US-897, and US-942 potentially viable feedstock sources for citrus co-products in the current HLB-blighted environment.

## 1. Introduction

Many citrus fruit types are grown worldwide and have major commercial value as fresh fruit, juice, and complementary products. In many citrus production areas, Huanglongbing (HLB), caused by *Candidatus* Liberibacter asiaticus (CLas), has devastated citrus tree health and decimated citrus fruit production. In Florida, the production of citrus has declined by 75% since the first detection of HLB in Florida in 2005 [1]. Most commercially grown citrus fruit types are severely affected by HLB. Methods to control CLas spread, such as tree removal and replacement, are only partially effective and too expensive for many producers [2]. Unlike most *Citrus* spp., the cross-compatible species *Poncirus trifoliata* and some of its hybrids with *Citrus*, are known to be highly tolerant of HLB and continue to effectively produce fruit even after trees become infected. Three such hybrid cultivars previously released by USDA, US-897, US-802, and US-942, have demonstrated tolerance to HLB [3,4,5,6,7] and can be grown in Florida long-term to produce viable crops with minimal pest and disease control.

Although first-generation hybrids of *Citrus* with *Poncirus* typically have fruit with undesirable flavor [8], they may be suitable for the production of many secondary processed products with high value. A number of products such as sugars, pectic hydrocolloids, limonene, volatiles, phenolics, and flavonoids come from citrus fruits that can serve as a chemical feedstock for the fuel, chemical, pharmaceutical, food and cosmetic industries [9,10,11]. Various methods to extract these compounds from citrus fruits have been explored including steam explosion (STEX) [9,12,13]. STEX is a hydrothermal process where steam at elevated temperature and pressure is applied to a material for a designated period followed by rapid decompression. This results in fragmentation of the material and an increase in the available surface area resulting in greater extraction yields. Intrinsic to STEX, limonene and other high-value volatiles can be removed and collected separately from the remaining by-products. STEX, as an extraction method, is inexpensive, environmentally friendly, and can be easily incorporated into existing citrus processing infrastructure where steam is already produced.

STEX was used initially as a pretreatment method for sweet orange residues and sugar beets to produce ethanol [14,15,16,17,18,19]. However, the conversion of citrus residues to ethanol does not maximize their value. Therefore, STEX was explored as a pretreatment method to extract and isolate high-value compounds within citrus residues. It was demonstrated that pectic hydrocolloids, sugars, phenolics, flavonoids, and volatiles from sweet orange and grapefruit residues could be obtained in high yields using STEX [18,20,21,22,23,24,25]. The total potential value that could be obtained from these citrus residues using STEX in a single season ranged between USD 70 and 760 million depending on the variety processed in Florida [20,21,22]. With the current HLB-blighted environment stymying citrus juice production, farmers can use the highly tolerant hybrids to produce a valuable chemical feedstock that serves a variety of industries. In this study, we investigated the quantities of pectic hydrocolloids, sugars, flavonoids, and volatiles that can be extracted and isolated from the citrus fruit varieties US-897, US-802, and US-942 using a continuous, pilot-scale STEX system and calculated their potential value.

## 2. Materials and Methods

### 2.1. Citrus Hybrid Fruit

**Figure 1:**
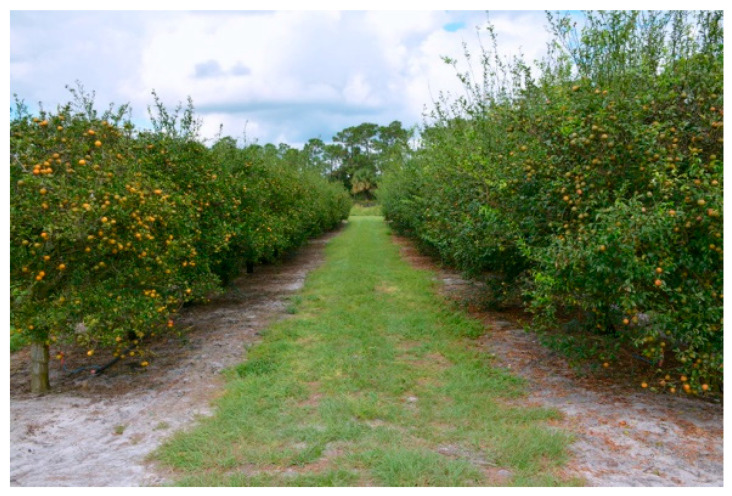
Fruiting trees in October 2019 of US-897 (left) and US-942 (right) used in the study.

The three hybrid cultivars US-802, US-897, and US-942 were chosen for the study because of observed field tolerance to HLB and the availability of a large number of fruiting trees [3,4,5,6,7]. The parentage of the hybrids is a follows: US-802 (*Citrus maxima* × *Poncirus trifoliata*), US-897 (*C. reticulata* “Cleopatra” × *P. trifoliata*), and US-942 (*C. reticulata* “Sunki” × *P. trifoliata*). Fruit were harvested from multiple 15-year-old trees of each selection growing at the USDA Picos Farm in Ft. Pierce, Florida (Figure 1), in fall 2018 and 2019, and used for components of the study as described below. Trees were grown with a spacing of 3.0 m between trees down the row with 6.1 m between rows. Trees were irrigated as needed with microjets from good quality (total dissolved solids between 300 and 600 mg/L) well water. The trees received fertilization and insect control according to recommended practices for citrus cultivation in this area of Florida [26,27] with minimal insect and disease control. All of the sampled trees have been infected by CLas for at least ten years, since they were confirmed CLas positive in 2009–2010 by PCR. The development of the infection specifically in the US-897 trees from 2007 to 2009 (with PCR results) was described in the publication by Albrecht and Bowman [3].

Four individual fruiting trees of each cultivar were used for the measurement of fruit size and weight, number of fruit per tree, and canopy area in October 2019 (Table 1). US-897 and US-942 were grown grafted on US-802 rootstock, while US-802 was grown using Sun Chu Sha mandarin as the rootstock. These scion rootstock combinations were compatible, but no evaluation was made of relative production on other rootstocks. Fruit yield per hectare was calculated from the actual tree density of 539 trees per hectare. The average percent juice, seed, and peel for 14–15 pieces of each fruit variety harvested in November 2020 were also measured (Table 1).

**Figure 2:**
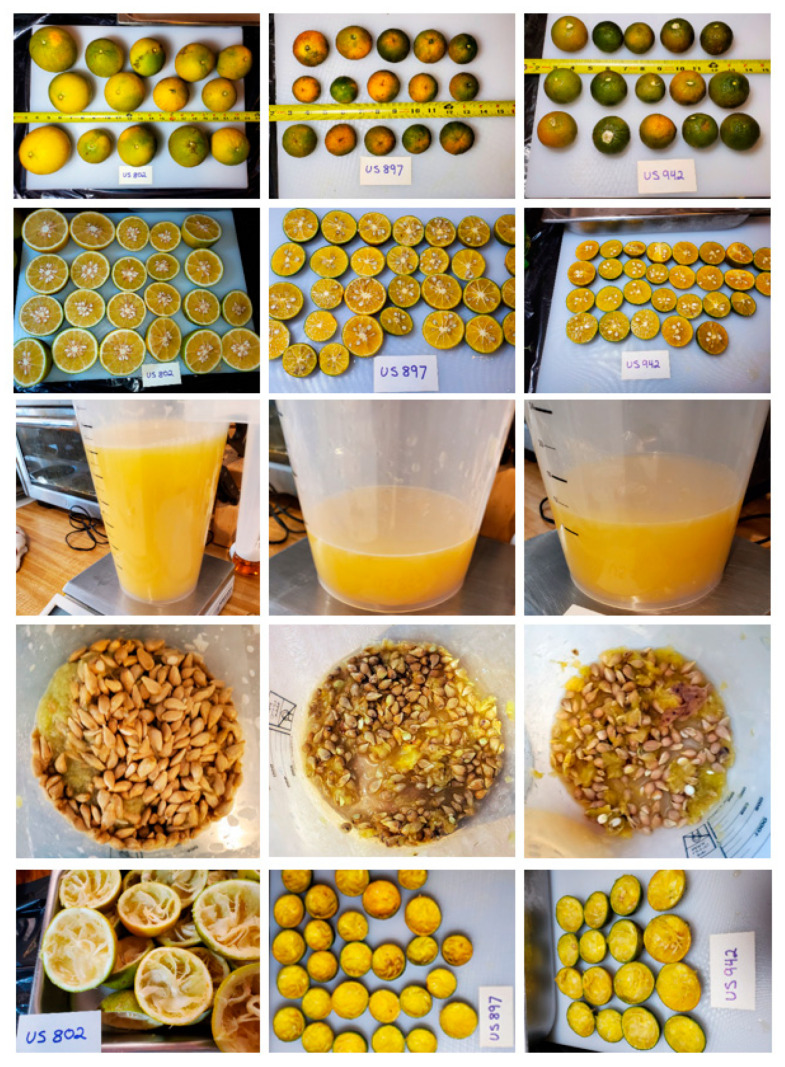
Whole fruit and the fractions collected from juicing (juice, seed, peel) US-802, US-897, and US-942 harvested November 2020.

For each variety, the mass of each piece of fruit was recorded. Whole fruit were cut and juiced by hand using a glass juicer or Proctor Silex Alex’s Lemonade Stand juicer (Model# 66331, Hamilton Beach Brands, Glen Allen, VA, USA). The mass of the juice, seed, and pulp was determined for each variety and the percentages of these fractions with respect to the total weight of all the fruit for a single variety were calculated. The remaining percent was designated as peel. Images of the fruit and their fractions are shown in Figure 2.

### 2.2. Harvesting and Preparation of Citrus Hybrid Fruit

Fruit samples of US-897, US-942, and US-802 were harvested in November 2018 and placed in large plastic bins. The bins of harvested fruit were placed in cold storage (5 °C) for approximately two weeks. The fruit was removed from cold storage and cleansed by soaking in a mixture of bleach and soap for approximately 5 min followed by rinsing. Fruit were then placed in clean plastic bins at room temperature overnight. Clean, dry, and mature fruit were cut open with a knife and seeds were removed. The remaining part of the fruit was collected in buckets and size reduced using a knife. This made up the majority of the citrus hybrid residues used in this study. Fruit not suitable for seed extraction were simply size reduced and collected in the same buckets. The citrus hybrid residues were weighed and stored at room temperature for no more than 2 h prior to STEX.

### 2.3. Continuous Pilot-Scale STEX of Citrus Hybrid Fruit

A continuous pilot-scale STEX system, described previously [21], was used to process the citrus hybrid residues. Briefly, residues were manually fed from the buckets into a hopper. An auger at the base of the hopper conveyed the residues to a jet cooker that injected saturated steam into the residues. The residues were treated with saturated steam at approximately 135 °C for 1–3 min in a hold tube with a pneumatic valve at the end. The pneumatic valve was set to open in order to maintain a pressure of 50 psi in the system. A manual pressure relief valve located in parallel to the pneumatic valve was manually opened only if the pressure approached the high set point of 75 psi. Upon opening the valve, steam-treated residues were fragmented and collected in a flash tank. The water vapor and volatiles released upon fragmentation were condensed and collected in a separate tank. The hot fragmented citrus hybrid residues were conveyed using a high-solids pump and collected in plastic-lined buckets throughout the process and allowed to cool and then frozen (−20 °C). Once all the citrus hybrid residues had been processed and collected, the system was shut down. The water vapor and volatiles were allowed to cool overnight in the tank to allow the formation of an upper organic layer containing the volatiles and a lower aqueous layer of the condensed water vapors. A schematic of the continuous pilot-scale STEX system was published previously [21]. The volatiles were collected separately from the aqueous layer in a glass bottle, sealed, and placed in cold storage (4 °C). Samples of the citrus hybrid residues before and after STEX were dried to a constant weight at 70–75 °C and dry weight measurements were completed in triplicate. The STEX parameters for each citrus hybrid and average dry weights before and after STEX can be found in Table 2.

### 2.4. Peel Oil Content

Peel oil content was determined as described previously [21] using the Scott bromate titration method [28]. Averages of triplicate analysis and the corresponding standard deviations are reported.

### 2.5. Soluble and Compositional Sugar Content

Soluble and compositional sugar content was determined as described previously [21]. Briefly, Fresh or STEX citrus hybrid material (25–50 g) were diluted in deionized water to 25% (*w/v*) and size reduced in a Waring Commercial Blender (Model# 7011S). Compositional sugars (glucose and fructose) and galacturonic acid were determined by enzymatic hydrolysis of 10 g of the size reduced and diluted samples in 1.25 mL of 50 mmol L^−1^ sodium acetate buffer, pH 4.8 and 1.25 mL of deionized water, using 100 µL each of two different pectinases (DSM, PAC, Batch 16B04V1, pectinase activity, 49.43 U mL^−1^, and Rapidase PNS, pectinase activity, 58.29 U mL^−1^), 50 µL cellulase (Novozyme, Cellic CTec2, VCPI0003, cellulase activity, 208.21 FPU mL^−1^), and 50 µL β-glucosidase (Novozyme 188, DCN00205, β-glucosidase activity, 270.67 U mL^−1^) with rotation for 24 h at 45 °C. To prevent microbial growth, 37 µL of cycloheximide (5 mg mL^−1^ stock) and 37 µL of chloroamphenicol (10 mg mL^−1^ stock) were added. Samples were then filtered prior to analysis to remove insoluble solids using a 0.45 µm GD/X Nylon syringe filter. The water extract of diluted samples was prepared for soluble sugars (glucose, fructose, and sucrose) analysis by removing insoluble solids by filtration, using a 0.45 µ GD/X Nylon syringe filter. Soluble and compositional sugars were quantified and identified by direct high-performance ion-exchange chromatography (HPIEC) analysis of the clarified extracts [21].

### 2.6. Density and GC/MS Analysis of Condensed Volatiles

The density and gas chromatography coupled with mass spectrometry analysis of the organic layer of condensed volatiles that were collected during STEX of each of the citrus hybrids were conducted as before [21].

### 2.7. Acid Extraction of Pectin from Fresh Peel

Fresh citrus fruit peel from the hybrids described above were cut into approximately 1–3 cm pieces. In a 1 L jacketed reaction vessel, 150 g of fresh peel was added to 650 mL of deionized water preheated to 70 °C. The pH was adjusted to 1.8 using concentrated nitric acid (HNO_3_). The slurry was stirred for 3 h, after which the pH was raised to pH 2.2 with 5 M sodium hydroxide (NaOH). The slurry was filtered through two layers of cheesecloth. Two volumes of propan-2-ol were used to precipitate the solubilized pectin, overnight at room temperature. The precipitate was centrifuged at 15,000× *g* for 20 min at 20 °C. The pectin pellets were frozen at −20 °C and then lyophilized (FreeZone Freeze Dry System; Labconco, Kansas City, MO, USA).

### 2.8. Recovery of Pectic Hydrocolloids

Pectic hydrocolloids were recovered from the STEX biomass using a simple water wash [22,25]. STEX biomass was mixed with an equal weight of deionized water (100 g each, three replicates per sample) and placed on a wrist shaker for 30 min at room temperature. Following centrifugation at 15,000× *g* for 20 min at 4 °C the supernatant was recovered, and the pellet was washed two more times as described above. The carbohydrate composition of each wash was determined via enzymatic hydrolysis and HPLC—PAD, in duplicate [23]. The three wash supernatants were pooled and preserved (0.02 wt% lithium azide), and then stored at 4 °C. Filtration through 1.2 μm glass filter fiber (GF/C, Whatman/GE Healthcare Life Sciences, Ltd., Chalfont Saint Giles, UK) was used to remove residual solids. Pectic hydrocolloids contained within the pooled supernatants were precipitated with acidified ethanol (55% final concentration) overnight at room temperature [29]. The precipitated pectic hydrocolloids were centrifuged at 15,000× *g* for 20 min at 20 °C. The pellets were washed in acidified ethanol and centrifuged as described above. The washed pellets were lyophilized and stored at −80 °C.

### 2.9. Macromolecular Characterization of Pectic Hydrocolloids

Size exclusion chromatography, coupled to a Multiangle Laser Light Scattering photometer, a differential pressure viscometer, and a differential refractive index detector (Wyatt Technology, Santa Barbara, CA, USA) was used to determine number average (M_n_), weight average molecular weight (M_w_), and intrinsic viscosity [η] [25].

The degree of methylesterification (DM) of the pectins was determined according to Ferguson et al. (2021). A 100 µL aliquot of 0.2% pectin solution was mixed with 470 µL of 25 mM acetate buffer and 30 µL of desalted PAC Rapidase fiber enzyme (DSM, Herleen, The Netherlands) in a 1.5 mL centrifuge tube. The pectin samples were incubated for 24 h at 40 °C. The pectin digests were then removed from the incubator and analyzed for the methanol and galacturonic acid (GalA) concentrations. Briefly, the GalA concentration was determined by first preparing a buffered copper solution containing 23.2 g NaCl, 2.3 g sodium acetate, and 1.0 mL glacial acetic acid to 80 mL of DI water. After the solutes were dissolved, 0.5 g CuSO_4_ was added, the pH was adjusted to 4.8 using 1 M NaOH, and the final volume was brought to 100 mL. A total of 50 µL of the buffered copper solution, 6 µL of a 0.2% (*w/v*) pectin digest solution, and 44 µL of pH 4.8 sodium acetate buffer per well were mixed in a clear polystyrene 96 well plate (Part No. 3903, Corning Inc., Corning, NY, USA). The microplate was then sealed with a microplate adhesive seal and was heated at 90 °C for 60 min. The microplate was then removed from the heat and 200 µL of solution “A” containing 5.0 mM bicinchoninic acid in pH 10.1 carbonate buffer was added. The absorbance of the GalA was measured at 550 nm [30,31,32]. The methanol concentration was determined using a previously reported Purpald (4-Amino-3-hydrazino-5-mercapto-1,2,4 triazole) method [33] with the following modifications, 6 µL of the 0.2% digested pectin solution was added to 84 µL of 200 mM pH 7 phosphate buffer in a 96 microwell plate. Ten µL of a dilute 0.005 U/mL Alcohol Oxidase (Product No. A2404, Sigma Aldrich, St. Louis, MO, USA) solution was added to each well. The reaction was allowed to proceed for 60 min at room temperature to ensure complete conversion of methanol to formaldehyde [34,35]. The formaldehyde concentration in each well was measured by adding 100 µL of a 34 mM Purpald solution, mixed into each well, and allowed to develop at room temperature for 30 min in the dark. A 100 µL solution of 33 mM NaIO_4_ was used to quench the Purpald reaction then the solution absorbance was determined at 550 nm [30]. The DM of pectin was calculated using Equation (1).

(1) $$DM=\frac{\left(Concentration\;of\;MeOH\right)}{\left(Concentration\;of\;GalA\right)}\times 100$$

Degree of branching (DBr; Gal + Ara/Rha) and the GalA/Rha ratio were calculated using weight % from the compositional sugar analysis [36].

(2) $$DBr=\frac{\left(Galactose+Arabinose\right)}{\left(Rhamnose\right)}$$

The HG/RG ratio was calculated using Equation (3). This is a better approximation of the HG/RG ratio due to a 1:1 ratio of GalA and rhamnose being present in the RG section of pectin; removing this GalA from the sum of the GalA concentration leads to a better HG/RG approximation.

(3) $$\frac{HG}{RG}ratio=(\frac{Galacturonic\;Acid-1/2\left(Rhamnose\right)}{Rhamnose})$$

For M_w_, polydispersity index and [η] three replicates were run for each sample. For DM, four replicates were performed and for GalA%, GalA/Rha, and DBr, two replicates were analyzed. The data were statistically analyzed by two-way ANOVA and Tukey’s Multiple Comparison (GraphPad Prizm, version 9.0.1 for Windows, GraphPad Software, San Diego, CA, USA).

### 2.10. Flavonoid Content

Flavonoid identification and quantification were conducted as described previously [21]. Briefly, methanol and water extracts of fresh and STEX citrus hybrid material were subjected to high-performance liquid chromatography-photodiode array mass spectrometry (HPLC-PDA-MS) analysis. Water extracts were prepared by taking a 1:3 ratio of deionized water to fresh and STEX citrus hybrid material and homogenizing and or blending (Omni International homogenizer, Model GLH-01, Omni International, Marietta, GA, USA or Waring Commercial Blender, Model# 7011S, Waring Commercial, Tarrington, CT, USA). The samples were microcentrifuged, and the supernatants were used for analysis. Methanol extracts were prepared by taking 1.5 g of fresh citrus hybrid material and homogenizing (Omni International homogenizer, Model GLH-01, Omni International, Marietta, GA, USA) in approximately 30 mL of methanol. The sample was then vacuum filtered. This was repeated two times more, and the filtered extracts were pooled and brought to 100 mL volume with methanol. A 15 mL portion was dried in a concentrator (SpeedVacConcentrator, SVC 200H, Thermo Scientific, Waltham, MA, USA) and brought to 4 mL with dimethylsulfoxide and was used for analysis.

## 3. Results and Discussion

### 3.1. Soluble and Compositional Sugars

For this study, the amount of glucose, fructose, and sucrose that can be extracted from size-reduced fresh or steam treated material with water are defined as soluble sugars. The amounts of glucose and fructose that can be extracted from size-reduced fresh or steam treated material with water after enzymatic hydrolysis are defined as compositional sugars and can be treated as total available glucose and fructose due to enzymatic breakdown of cellulose, cellobiose, hemicellulose, and sucrose. The soluble and compositional sugars of fresh and steam-treated US-802, US-897, and US-942 residues are shown in Table 3. For comparison, the soluble and compositional sugars of fresh Valencia and Hamlin oranges, as well as Marsh grapefruit and a mixture of Ruby, Star, and Flame grapefruit citrus processing residues are also listed in Table 3.

All three citrus hybrids, when fresh, have similar amounts of soluble glucose, fructose, and sucrose to that of fresh Valencia. Fresh US-802 and US-897 have similar amounts of compositional glucose and fructose to that of fresh Valencia. Fresh US-942 has much less of both monosaccharides. The amounts of water-soluble glucose and fructose for all three hybrids increased because of the breakdown of sucrose by STEX. STEX and enzymatically hydrolyzed US-897 produced the greatest amount of glucose and fructose among the citrus hybrids tested. Ultimately, STEX led to modest increases in glucose and fructose in all three citrus hybrids with and without enzymatic hydrolysis except in the case of the hydrolyzed and steam-treated US-802 sample. While the use of enzymes resulted in an increase in the amounts of glucose and fructose that could be extracted from the citrus hybrids, STEX alone was almost as effective. In fact, STEX alone was capable of releasing 49–62% of the total available glucose and 67–93% of the total available fructose for extraction.

### 3.2. Peel Oil, Density, and Chemical Composition of Condensed Volatiles

Citrus essential oils, mainly derived from peel tissues, are made up of 85–99% semi-volatile and volatile compounds and are found primarily in the flavedo [37]. US-942 had the greatest amount of peel oil, followed by US-897 and US-802 (Table 4).

STEX released 53, 83, and 85% of the peel oil found in US-802, US-897, and US-942, respectively. This is based on the difference in the amount of peel oil in fresh citrus hybrid tissue and STEX citrus hybrid samples. These calculations can be seen in the Supporting Information section. In the process of STEX, the citrus peel oil volatiles of US-942, US-897, and US-802 were condensed and collected. These volatiles are typically composed of 60–95% limonene [37] so densities should be close to the density of limonene (0.841 g/mL). All three citrus hybrids had less D-limonene, octanal, nonanal, and decanal than what is observed in the volatiles of major Florida citrus varieties, but the densities of US-942 and US-897 are still similar to that of limonene (Table 5). The chemical composition of essential oils can vary depending on plant health, growth stage, climate, soil conditions, harvest time, processing conditions, distillation, storage, and handling [39]. The presence of heat, light, and oxygen can lead to isomerization, oxidation, dehydrogenation, polymerization, and thermal rearrangements [39]. US-802 has a significantly greater density and a greater concentration of oxygenated compounds, specifically caryophyllene oxide when compared to US-942 and US-897 (Table 5). Caryophyllene oxide serves as a broad-spectrum antifungal in plant defense and as an insecticidal/antifeedant [40,41]. All three citrus hybrids had more β-myrcene and (E)-caryohphyllene than what is observed in other Florida citrus varieties (Table 5). US-942 and US-897 had more β-pinene than US-802 and what is observed in other Florida citrus varieties. Citrus peel oil is a high-value secondary product of citrus juice processing with orange oil currently selling for USD 4.5/kg and grapefruit oil selling for up to USD 65/kg [42]. This is due to its use in a variety of products in the food, cosmetic, pharmaceutical, and chemical industries. Peel oil is extracted at various stages in the juice processing plant [43].

The amount of oil extracted from peel, as well as its composition, are dependent on the variety, cultivation, extraction, and purification methods employed [45]. Valencia and Hamlin sweet orange fruit, the major varieties used for producing orange juice, can contain approximately 0.4% and 0.7% (*w/w*) peel oil, respectively [46,47]. Marsh and Ruby grapefruit varieties can contain 0.31 and 0.325% (*w/w*) peel oil, respectively [47]. US-802, US-897, and US-942 were found to have 0.13, 0.48, and 0.69% (*w/w*) peel oil, respectively (Table 4). Recently, studies have focused on the effect of HLB on the various products derived from citrus fruits [44,48]. In a study investigating the effects of HLB on the flavor and aroma of cold-pressed oils from Hamlin and Valencia oranges, panelists described asymptomatic late-season Valencia as sweeter smelling and symptomatic Hamlin and Valencia as sour smelling [44]. Another study found that the amount of juice oil in HLB affected orange juice was lower compared to that derived from healthy fruit and the chemical profile of the volatiles differed greatly. Specifically, peel oil was found to contain lower quantities of aldehydes, which are associated with the normal ripening of orange fruits [48].

### 3.3. Recovery and Macromolecular Properties of Pectic Hydrocolloids from STEX

**Figure 3:**
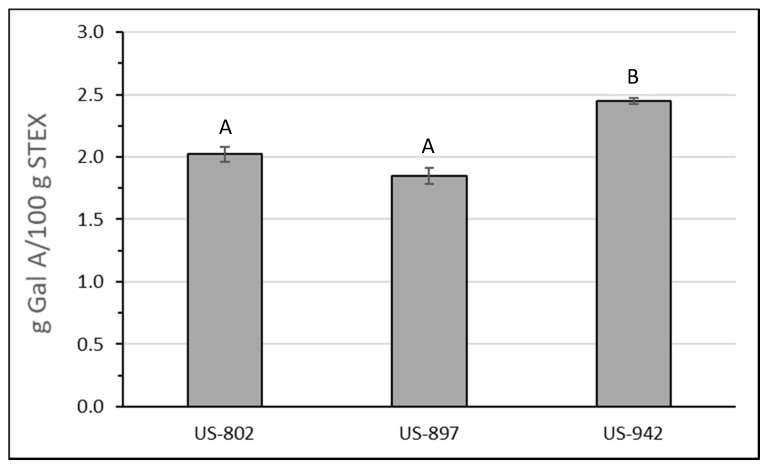
Recovery of pectic hydrocolloids from steam-exploded (STEX) hybrid peel as determined by the amount of galacturonic acid in wash supernatants after normalizing for % dry wt. Error bars represent the standard error of the mean. Bars with different upper case letters indicate a statistically significant difference (*p* < 0.05) between hybrid groups.

Figure 3 shows the recovery of pectic hydrocolloids, as estimated by the recovery of GalA, following STEX of the three different citrus hybrids. The highest recovery was from US-942. Recovery amounts for all three hybrids were higher than previously observed for Hamlin orange peel and within the range observed from Valencia orange peel [22].

**Figure 4:**
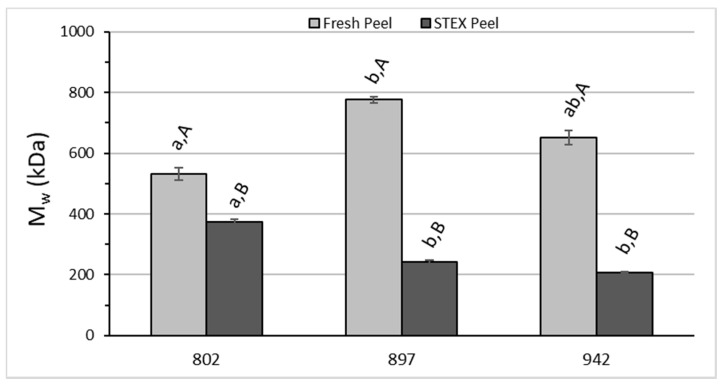
Weight average molecular weight (M_w_) of recovered pectic hydrocolloids from acid extracted fresh peel and a water wash of steam-exploded (STEX) peel. The absence of error bars indicates the standard error of the mean was too small to be visible. Bars with different lower case letters indicate a statistically significant difference (*p* < 0.05) between hybrid groups for either acid extraction from raw peel or steam explosion. Bars with different upper case letters indicate a statistically significant difference (*p* < 0.05) within a hybrid group for each type of extraction.

**Figure 5:**
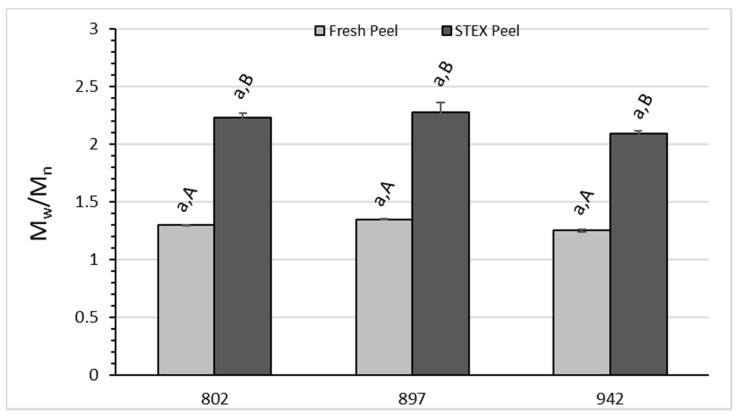
Polydispersity index (M_w_/M_n_) of recovered pectic hydrocolloids from acid extracted raw peel and a water wash of steam-exploded (STEX) peel. The absence of error bars indicates the standard error of the mean was too small to be visible. Bars with different lowercase letters indicate a statistically significant difference (*p* < 0.05) between hybrid groups for either acid extraction from raw peel or steam explosion. Bars with different uppercase letters indicate a statistically significant difference (*p* < 0.05) within a hybrid group for each type of extraction.

**Figure 6:**
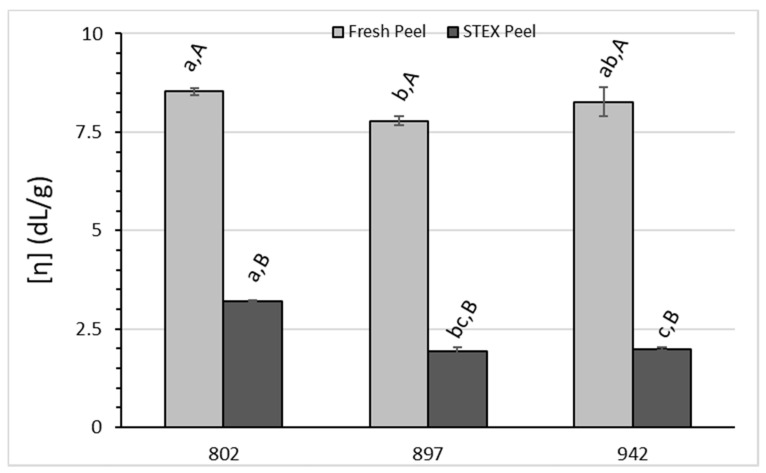
Intrinsic viscosity [η] of recovered pectic hydrocolloids from acid extracted raw peel and a water wash of steam-exploded (STEX) peel. The absence of error bars indicates the standard error of the mean was too small to be visible. Bars with different lowercase letters indicate a statistically significant difference (*p* > 0.05) between hybrid groups for either acid extraction from raw peel or steam explosion. Bars with different uppercase letters indicate a statistically significant difference (*p* > 0.05) within a hybrid group for each type of extraction.

The average molecular weight (M_w_), polydispersity index (M_w_/M_n_), and intrinsic viscosity ([η]) for acid extracted and STEX pectic hydrocolloids were analyzed by two-way ANOVA (Figure 4, Figure 5 and Figure 6). M_w_ values reported here (Figure 4) for acid extracted pectin are higher than what is generally found for pectin from lemon, lime, or orange fruit peel [49]. M_w_ of the STEX peels are more like the values reported for these other citrus fruits. For M_w_ the analysis indicated statistically significant effects due to hybrid source (*p* = 0.0074), extraction method (*p* < 0.0001), and the interaction between hybrid and extraction method (*p* < 0.0001). Significant differences for M_w_ between extraction methods were observed for each hybrid (Figure 4). Significant differences were also observed among hybrids for pectic hydrocolloids obtained by acid extraction and STEX. Using the acid extraction method, US-802 differed from US-897 (*p* = 0.003), but US-942 was not significantly different from either US-802 or US-897. For pectic hydrocolloids obtained following STEX, US-802 was significantly different from both US-897 and US-942 (*p* < 0.05 and *p* = 0.0055, respectively), but US-897 and US-942 did not differ significantly. The observed values for M_w_ in this study are in line with values reported by [22] for the static STEX of var. Hamlin and Valencia, and for continuous STEX of pre-harvest dropped or processor culled HLB fruit peel [23,24], but larger than values reported by [25] for continuous STEX of var. Hamlin or Valencia fruit peel.

For the polydispersity index, there were statistically significant effects due to the extraction method (*p* < 0.0001), but there was no significant difference for hybrid source or the interaction hybrid × extraction method. Polydispersity values observed for the acid extracted pectins (Figure 5) were within the range reported by [50] for microwave extracted lime pectins. The polydispersity index for pectic hydrocolloids obtained with the STEX of these citrus hybrids were lower than previously observed from STEX [23,25]. The polydispersity index for either acid extracted or STEX pectic hydrocolloids was not statistically different for any hybrid group within an extraction method. However, within each hybrid group, the polydispersity of the acid extracted pectin was significantly lower than the STEX material (*p* < 0.0001 for each hybrid group). Polydispersity values reported here for the STEX biomass are much lower than those reported for juice extracted sweet orange peel or pre-harvest dropped Hamlin sweet oranges [24,25] but in agreement with those reported for HLB infected sweet oranges [23].

The [η] analysis (Figure 6) leads to the conclusion that there are statistically significant effects due to the hybrid source (*p* < 0.01) and extraction method (*p* < 0.0001), but the interaction of the hybrid x extraction method was not significant (*p* = 0.0519). Tukey’s multiple comparison test indicated some significant differences for the pectic hydrocolloids from the different hybrids and extraction methods. In each of the hybrid groups, [η] was always higher for acid extraction vs. STEX (*p* < 0.01). This has been observed in several studies comparing pectic hydrocolloids from acid extraction vs. STEX [23,24] and the lower values for pectic hydrocolloids from STEX biomass have been shown to decrease with both temperature and time-at-temperature during STEX [22]. Within the acid extracted group, US-802 was significantly different from US-897 (*p* = 0.0025) and US-942 was not significantly different from either US-802 or US-897. Within the STEX group, US-802 differed from both US-897 and US-942 (*p* = 0.0177 and *p* = 0.0048, respectively), but US-897 and US-942 did not differ statistically from each other (*p* = 0.753).

The effect of hybrid group, or extraction method, on the DM, GalA%, GalA/Rha ratio, and DBr of pectic material recovered by either a water wash of STEX peel or an acid extraction of fresh peel, are listed in Table 6.

Two-way ANOVA of DM for STEX peel indicated no significant difference between US-802 and US-897 or US-942, but in fresh peel, US-897 and US-942 were significantly different from each other (*p* < 0.05). Method of extraction also produced pectins with significantly different DMs for US-897 and US-942 (*p* < 0.0001). The amount of pectic hydrocolloids recovered by a water wash from the STEX peel (Figure 3) represents 84.4%, 82.8%, and 81.3% of the pectin present in US-802, US-897, and US-942 fruit peel, respectively. The higher DM values observed for these STEX pectic hydrocolloids, compared to acid extracted pectin from fresh peel, suggests the insoluble pectic materials had a sufficiently low DM to reduce the population level DM to the observed values. Another possibility is that the STEX process causes a loss of HG methyl esters.

A GalA % value of greater than 65% on a moisture, sugar, and ash-free basis is required for a material to be defined as pectin according to the [51]. Table 6 indicates that this condition is met by all the pectins that were acid extracted from fresh peel, but not for the STEX US-897 and US-942 material, which could possibly be differently labeled (i.e., pectic hydrocolloids). In both US-897 and US-942, the GalA % in the STEX extracted material was statistically less than in the acid extracted, fresh peel pectin. Within the fresh peel pectins, US-897 differed from US-942 (*p* < 0.05), and within the STEX group, there were no significant differences. The values reported here are lower than values of 78% to 87% reported for pectin extracted from healthy and HLB grapefruit peel [52] or GalA % values from HLB and healthy Valencia sweet oranges (77.5% and 82.7%, respectively [53].

The GalA/Rha ratio is indicative of the ratio of the HG domains and RG I domains. Smaller ratios suggest either an increased number of RG I domains or a shortened HG stretch between larger-sized RG I domains. Previous comparisons of the effect of HLB on this parameter showed a decrease in the ratio for pectin from mature HLB grapefruit compared to pectin from healthy grapefruit [52]. The ratios were higher for all hybrid groups in the acid extracted pectin from fresh peel (Table 6), and like values reported for pectin from orange, lime, and grapefruit following a harsh HNO_3_ extraction [36]. These values are much lower than those reported for pectin extracted from healthy or immature HLB grapefruit peel, and closer to values from mature HLB grapefruit peel [52]. The ratio from the US-942 STEX peel was lower than either US-802 or US-897.

DBr is a measure of the amount of neutral sugar (Gal and Ara) branching off of Rha in the RG I domains of pectin. Pectins with lower DBr values have been shown to produce weaker Ca^2+^ gels [54]. As observed for GalA/Rha ratios, the DBr of all of the STEX pectins were lower than their acid extracted counterparts (Table 6). No significant differences were observed within the acid extracted fresh peel pectins, but again, the STEX US-942 DBr was significantly lower than the other two STEX samples.

Molecular weight, degree of methylesterification, and the distribution of charge within the homogalacturonan region are the three macromolecular properties reported to be responsible for pectin functionality [55,56]. M_w_ from STEX peel was lower than acid extracted pectin from fresh peel. The increase in polydispersity and decrease in [η] following STEX suggests these pectic hydrocolloids could have potential applications beyond pectin’s traditional use in food formulation [57,58,59,60].

A theoretical yield for pectic hydrocolloids from each of these hybrids that could be recovered following a conventional hot acid extraction from fresh peel was estimated based on the yield of fresh fruit per hectare, and the recovery amounts from STEX. The greatest yield of pectin per hectare was 0.10 MT for US-942, followed by 0.08 MT for US-802, and 0.04 MT for US-897. Based on GalA recoveries from the STEX processed peel, the greatest STEX yield per hectare was for US-942 (0.059 MT), followed by US-802 (0.055 MT) and then US-897 (0.024 MT).

### 3.4. Flavonoids

There are three classes of phenolic compounds with potential commercial value in the peels of fruit of the three citrus hybrids included in this study. These compound classes include flavanone glycosides, polymethoxylated flavones (PMFs), and coumarins. Individual compounds in each of these classes were identified or partially characterized by HPLC coupled with UV absorbance and mass spectrometry (HPLC-PDA-MS). Of course, not only are the individual classes of compounds of commercial interest but unique, targeted mixtures are presented which are not normally present in peels in the major processed citrus fruits.

#### 3.4.1. Flavanones

Flavonoids are the most abundant phenolics in the peels of citrus fruits (Massenti et al., 2016). Flavanone glycosides in citrus occur mainly as disaccharides of either neohesperidose (2-*O*-α-L-rhamnosyl-D-glucose) or rutinose (6-*O*-α-L-rhamnosyl-D-glucose) [61,62]. Among the sweet oranges (*C. sinensis*), mandarins (*C. reticulata*), and lemons (*C. limon*), the flavanone glycosides occur exclusively as rutinosides [63]. Whereas in other *Citrus* species, grapefruit (*C. paradisi*), pummelo (*C. maxima)*, and the *Citrus* relative trifoliate orange (*Poncirus trifoliata*), the flavanone glycosides occur as neohesperidosides [63] The two mandarin × *Poncirus* crosses (US-842 and US-897) show sharply contrasting flavanone glycosides profiles (Table 7). U.S. 897 contains a high concentration of poncirin, this being a characteristic of its *Poncirus* parent, whereas U.S.942 contains a high concentration of neohesperidin. In contrast, hybrid U.S. 802 contains a high concentration of naringin. In this way, all three hybrids contain contrasting flavanone glycoside profiles.

The abundance of neohesperidin in US-897 and US-942 can be useful as a potential source of the flavanone glycoside for the production of the manufacture of the artificial sweetener neohesperidin dihydrochalcone and related compounds [64]. Flavonoid compounds from *P. trifoliata* can serve as potential candidates for use in the development of commercial mosquitocidal products that may be an alternative to conventional synthetic chemicals, particularly in integrated vector control applications [65]. Neohesperidin and poncirin have been investigated for their effect on gastritis in rats and human gastric cancer cells. Extracts rich in these compounds exhibit acid-neutralizing capacities and cytotoxicity against human AGS gastric cancer cells. Neohesperidin and poncirin significantly inhibit HCl/ethanol-induced gastric lesions and increase the mucus content [66]. Another study revealed that neohesperidin could inhibit allergic responses in vivo and in vitro, and that the molecule may be regarded as a novel agent for preventing mast cell-immediate and delayed allergic diseases [67]. Neohesperidin administration attenuated weight gain, low-grade inflammation, and insulin resistance in mice fed a high-fat diet (HFD). Neohesperidin administration substantially restored gut barrier damage, metabolic endotoxemia, and systemic inflammation. More importantly, it was demonstrated that the regulation of obesity could be transferred from neohesperidin-treated mice to HFD-fed mice via fecal microbiota transplantation [68].

#### 3.4.2. PMFs

PMFs have been extensively studied for their anticancer, anti-inflammatory, antilipogenic, antiobesity, and antidiabetic actions in animals [69,70,71,72,73], and are already commercialized as health-benefiting compounds. Table 7 compares the levels of tangeretin and nobiletin in the two mandarin × *Poncirus* crosses against recovered amounts of these compounds from fresh and STEX Valencia and Hamlin orange peels [22]. The hybrid US-942 exhibited twice the recoverable amounts of nobiletin and tangeretin compared to US-897. As expected, the hybrid US-802 contained none of the polymethoxylated flavones. US-942 contained less than half of the nobiletin of Hamlin and Valencia peels, but three times as much tangeretin as Hamlin and Valencia.

#### 3.4.3. Coumarins

US-802 hybrid represents a cross between pummelo (*C. maxima*) and *Poncirus trifoliata*. An obvious characteristic of this hybrid is the numerous coumarins in the fruit peel. These compounds were identified as coumarins based on their characteristic coumarin-like UV spectra and by their characteristic mass spectra fragmentation profiles. Although most of these compounds remain unidentified, five of the main coumarins were identified, including umbelliferone, auraptene, osthole, marmin, and meranzin. Identifications were made based on HPLC-MS exact peak overlaps with authentic standards (data not shown). Low molecular weight prenylated coumarins, similar to those found in the US-802 hybrid, possess potent anti-inflammatory and anti-HIV activities in vitro [74,75]. Osthole, a minor prenylated coumarin constituent in US-802 demonstrates multiple pharmacological actions including neuroprotective, antimicrobial, antiviral, immunomodulatory, anticancer, hepatoprotective, and cardiovascular protective activities. Activities of osthole are very likely linked to its influence on cyclic adenosine monophosphate (cAMP) and cyclic guanidine monophosphate (cGMP) levels [76]. Osthole also shows antiviral activity [77]. Marmesin has been known to exert antitumor activity against several types of cancer cells including colon cancer [78,79] and structurally related meranzin hydrate induces anti-atherosclerosis and behavioral improvements in high-fat diet ApoE−/− mice via anti-inflammatory and BDNF-TrkB pathway [80]. Therefore, there are many potential commercial uses for the coumarins recoverable in U.S. 802 extracts.

### 3.5. Potential Market Value

The amount of the value-added compounds that could be recovered from US-802, US-897, and US-942 were calculated using the mass per hectare and the percent peel data in Table 1 along with the amount of each value-added compound that could be recovered using STEX (Table 3, Table 4 and Table 7 and Figure 3). The results of these calculations can be seen in Table 8. These values were then used to estimate the potential value that could be earned from each variety based on the market value of various final products. These calculations can be seen in Table S1 of the Supplementary Information.

STEX is capable of releasing 49–62% of the total available glucose and 67–93% of the total available fructose for extraction without the addition of enzymes. The amount of glucose that could be recovered using STEX would be 323 kg/ha for US-802, 158 kg/ha for US-897, and 304 kg/ha for US-942. These sugars can serve as precursors for the production of bio-based commodity chemicals [81,82,83,84,85], increasing their overall value. For example, if we calculate the value of converting the glucose produced from STEX of each citrus hybrid to ethanol, this would result in a value of USD 517 per hectare of US-897, USD 1053 per hectare of US-802, and USD 994 per hectare of US-942 (See Table S1 of the Supplementary Information).

US-897 and US-942 contain oils in amounts similar to what is observed in other major Florida citrus varieties (Table 4). STEX is capable of releasing 83–85% of these volatiles that upon condensation give similar chemical profiles to other major Florida citrus varieties (Table 5). This equates to approximately 27 kg per hectare of oil from US-897 and 58 kg per hectare of oil from US-942 that could potentially be collected via STEX. If we assume a value similar to orange oil this equates to about USD 120 per hectare for US-897 and USD 259 per hectare for US-942 (See Table S1 Supplementary Information). They, therefore, have the potential to be grown and processed for their oil as a high-value product.

A theoretical yield for pectic hydrocolloids from each of these hybrids that could be recovered following a conventional hot acid extraction from fresh peel was estimated based on the yield of fresh fruit per hectare, and the recovery amounts from STEX. The greatest yield of pectin per hectare was 0.10 MT for US-942, followed by 0.08 MT for US-802, and 0.04 MT for US-897. The values associated with these yields can be estimated by using reported wholesale prices [86]. These prices range from USD 16.50 per kg for high methoxy pectin (DM > 50%) to USD 20.25 per kg for Rapid Set pectin. Assuming a price of USD 20 per kg, the per hectare value of the pectin recovered from these hybrids would be USD 2035 for US-942, USD 1522 for US-802, and USD 796 for US-897. The value of the pectic hydrocolloids recovered following STEX is harder to estimate since the structural/functional properties of the STEX pectin differ from the acid extracted pectin from fresh peel. However, the STEX pectic hydrocolloids might have potential use in higher value, non-food applications [87]. Based on GalA recoveries from the STEX processed peel, the greatest STEX yield per hectare was for US-942 (59 kg), followed by US-802 (55 kg), and then US-897 (24 kg). A rudimentary cost analysis considering the cost of industrial production of STEX pectic hydrocolloids is provided in Table S2 of the Supporting Materials. The cost analysis shows that the value of producing STEX pectic hydrocolloids is considerably less than the potential high-value products it could replace.

Of the compounds that were identified in each of the citrus hybrids, naringin, neohesperidin, and poncirin were the most abundant in US-802, US-942, and US-897, respectively. Using the values of fruit mass per hectare from Table 1 along with the amount of each compound present in each hybrid from Table 7, we calculated that the use of STEX can yield approximately 48 kg of naringin per hectare of US-802, 29 kg of poncirin per hectare of US-897, and 27 kg of neohesperidin per hectare of US-942. Naringin is valued at 30–65 USD per kilogram [88] and neohesperidin is valued at 15–90 USD per kilogram [89]. Poncirin is the most valuable of the three compounds with a value of 60,000–150,000 USD per kg [90], but these high prices for poncirin are likely due to the limited supply and market for this compound, and so a similar comparison with neohesperidin and naringin cannot be made. However, if it is presumed that poncirin has a true market value similar to its isomer, naringin, there are possible values of USD 1429–3096 per hectare of US-802, USD 399–2392 per hectare of US-942, and USD 1.7–4.3 million per hectare of US-897. The global flavonones market is currently valued at USD 114 million and is expected to grow to USD 141 million by 2027 [91]. The flavones found in US-802, US-897, and US-942 are just some of the many compounds that contribute to this market.

Another major source of economic value for these hybrids is the reduced cost of production compared to current commercial varieties [2,92,93]. Before HLB was widespread, the cost of maintaining a grove was USD 2869 per hectare and had risen to USD 4804 per hectare by the 2016–2017 season [92]. The cost of producing the hybrids would be less per hectare than oranges or grapefruit because special applications of fertilizer and pesticides related to HLB would not be needed.

## 4. Conclusions

The continuous, pilot-scale STEX of US-802, US-897, and US-942 followed by water extraction resulted in high yields of sugars, pectic hydrocolloids, volatiles, and flavonoids. Recoveries of these components were greatest on a mass per hectare basis among US-942 and US-802, with 323 kg of glucose per hectare from US-802, 58 kg of peel oil per hectare from US-942, 59 kg of pectic hydrocolloids per hectare from US-942, and 48 kg of naringin per hectare from US-802. Based on our calculations, there is potential monetary value in these components if they are converted to value-added products. The presence of coumarins from US-802, and neohesperidosides from US-897 and US-942 could make these hybrids very useful for various applications as precursors for food ingredients and as health-promoting compounds. Each hybrid has its own unique flavonoid profile that could be exploited depending on the final desired application. The innate tolerance to CLas and consequent ease of commercial production in HLB-endemic conditions, along with the potential value that can be earned from US-802, US-897, and US-942 makes them excellent candidates for citrus processing co-product feedstocks.

## Figures and Tables

**Table 1:** Fruit size and yield from 15-year-old trees of the three cultivars in a field planting in St. Lucie County, Florida.

Cultivar	Fruit Length (mm) ^1^	Fruit Diameter (mm) ^1^	Fruit Weight (g) ^1^	Fruit Juice (% wt, wet)	Fruit Seed (% wt, wet)	Fruit Peel (% wt, wet)	Fruit per Tree (kg) ^1^	Fruit per Hectare (kg) ^1^
US-802	88 ± 6	83 ± 7	217.1 ± 8.4	39.6	7.5	52.9	67.8 ± 3.8	36,500 ± 2100
US-897	41 ± 2	35 ± 3	29.8 ± 1.3	36.9	9.7	53.4	35.0 ± 6.1	18,900 ± 3300
US-942	41 ± 4	39 ± 3	38.7 ± 2.7	39.9	8.8	51.3	54.0 ± 8.0	29,100 ± 4300

^1^ Values are mean ± SD.

**Table 2:** US-802, US-942, and US-897 steam explosion (STEX) parameters and percent dry weight (% dw) before and after steam explosion (STEX).

Items	Total Mass Run (kg)	Average Temperature (°C)	Total Run Time (min)	% dw before STEX ^1^ (wt %, Dry)	% dw after STEX ^1^ (wt %, Dry)
US-802	133	135	92	16.36 ± 0.30	13.72 ± 0.20
US-897	82	134	27	16.25 ± 0.36	12.37 ± 0.05
US-942	79	135	34	20.69 ± 0.36	15.35 ± 0.04

^1^ Values are mean ± SD.

**Table 3:** Soluble and compositional sugar content (g/100 g dry) of fresh citrus hybrids and major Florida varieties and steam-exploded (STEX) citrus hybrids. Paired, two-tailed *t*-test *p*-values are given in parentheses.

Items	SOLUBLE					
	Fresh			STEX		
Variety	Glucose	Fructose	Sucrose	Glucose	Fructose	Sucrose
US-802	9.60 ± 0.13	8.49 ± 0.12	6.22 ± 0.18	11.86 ± 0.76 (0.04)	10.15 ± 0.71 (0.07)	2.29 ±0.17 (0.00)
US-897	10.06 ± 0.39	9.49 ± 0.37	6.31 ± 0.27	12.23 ± 0.26 (0.02)	11.08 ± 0.37 (0.04)	0.00 ±0.00 (0.00)
US-942	9.76 ± 0.50	9.09 ± 0.57	5.43 ± 0.34	12.62 ± 0.71 (0.01)	11.42 ± 0.66 (0.02)	0.00 ±0.00 (0.00)
Hamlin ^1^	12.21	13.85	7.37			
Valencia ^1^	9.58	8.86	7.86			
Marsh ^2^	13.69	12.71	9.81			
Ruby, Star, Flame ^2^	9.85	10.08	13.94			
	COMPOSITIONAL					
	Fresh			STEX		
Variety	Glucose	Fructose		Glucose	Fructose	
US-802	22.41 ± 0.99	11.37 ± 0.25		20.98 ± 1.42 (0.37)	10.95 ± 0.09 (0.09)	
US-897	22.39 ± 0.89	13.49 ± 0.32		25.11 ± 1.11 (0.00)	16.58 ± 0.11 (0.00)	
US-942	16.57 ± 1.54	10.26 ± 0.96		20.22 ± 0.89 (0.07)	13.35 ± 0.09 (0.03)	
Hamlin ^1^	24.05	16.18				
Valencia ^1^	20.94	12.98				
Marsh ^2^	27.25	18.89				
Ruby, Star, Flame ^2^	24.74	15.66				

^1^ Averages calculated from values for raw Hamlin or Valencia from Supplementary information [22]; ^2^ averages calculated from values for raw Marsh or a mixture of Ruby, Star, and Flame [20].

**Table 4:** Peel oil content of fresh and steam-exploded (STEX) citrus hybrids and percent of volatiles released. Paired, two-tailed *t*-test *p*-values are given in parentheses.

Items	Fresh (% *w*/*w*, Wet)	Fresh (% *w*/*w*, Dry)	STEX (% *w*/*w*, Dry)	Volatiles Released (%)
US-802	0.13 ± 0.00	0.79 ± 0.00	0.37 ± 0.01 (0.00)	53
US-897	0.48 ± 0.02	2.95 ± 0.13	0.51 ± 0.00 (0.00)	83
US-942	0.69 ± 0.04	3.32 ± 0.17	0.49 ± 0.04 (0.00)	85
Valencia [38]	0.7		-	-
Hamlin [38]	0.4		-	-
Ruby Red [38]	0.3		-	-
Marsh [38]	0.3		-	-

**Table 5:** US-802, US-942, and US-897 condensed volatile organic layer density and chemical composition as determined by GC/MS and compared to Florida Red Ruby, Marsh, Hamlin, and Valencia derived oils.

Reference				[21]	Deng et al. 2020	[44]	
Treatment	Steam Explosion			Steam Explosion	Molecular Distillation	Cold Pressed	
	De-Seeded Fruit			Brown Extracted Peel	Essential Oil	JBT Processed Fruit	
Variety	US-802	US-942	US-897	Ruby Red	Marsh	Late AS Hamlin	Late AS Valencia
Density (g/mL)	0.9263 ± 0.0003	0.8436 ± 0.0004	0.8452 ± 0.0003	0.8477 ± 0.0002	nr	nr	nr
Compound	% based on total ion current						
α-Pinene	1.01	0.82	0.75	0.36	0.76	0.50	0.48
β-Pinene	0.00	3.30	2.59	0.00 ^a^	0.05	nr	nr
β-Myrcene	4.63	3.39	3.81	1.66	2.16	1.38	1.21
Octanal	0.00	0.01	0.02	0.07	0.36	0.67	0.97
o-Cymene	1.66	0.20	0.19	0.06 ^a^	nr	nr	nr
D-Limonene	60.07	78.92	79.69	87.94	93.33 ^b^	96.6 ^b^	95.9 ^b^
Linalool	0.20	0.41	0.36	0.16	0.12	0.04	0.23
Nonanal	0.00	0.00	0.00	0.07	0.05	0.01	0.04
Decanal	0.00	0.02	0.02	0.07	0.19	0.09	0.29
Carvone	1.80	0.14	0.25	1.60	0.41	trace	0.01
Geranyl acetate	0.09	0.00	0.03	0.12	nr	nr	nr
α-Copaene	0.10	0.08	0.07	0.32	0.13	trace	trace
ß-Elemene	1.13	0.30	0.33	0.28	nr	nr	nr
(E)-Caryophyllene	6.89	2.18	3.13	0.89	0.20 ^c^	0.01 ^d^	0.02 ^d^
α-Humulene	1.11	0.28	0.36	0.15 ^a^	0.03 ^e^	trace	trace
δ-Cadinene	0.18	0.30	0.17	0.27	0.04	trace	nd
Caryophyllene oxide	7.66	0.26	0.36	0.43 ^a^	0.04	trace	0.01
Total	86.53	90.62	92.11	94.45	97.87	99.30	99.16
Oxygenated	9.75	0.84	1.04	2.52	1.17	0.81	1.55

AS = asymptomatic HLB fruit; nd = not detected; nr = not reported; ^a^ not reported in reference; ^b^ reported as Limonene; ^c^ reported as Caryophyllene; ^d^ reported as β-Caryophyllene; ^e^ reported as Humulene.

**Table 6:** Degree of methylesterification (DM), percent GalA, GalA/Rha, and degree of branching (DBr) of pectin extracted from STEX or fresh peel of citrus hybrids. Significance (*p* < 0.05), as determined by two-way ANOVA and Tukey’s multiple comparison test, within steam-exploded (STEX) or fresh groups is indicated by lower case letters. Significant differences within a hybrid group for extraction method are indicated by upper case letters.

Sample		DM (Mean ± SE)	Percent GalA (Mean ± SE)	GalA/Rha (Mean ± SE)	DBr (Mean ± SE)
STEX	US-802	60.34 ± 5.04 ^a,A^	69.46 ± 1.49 ^a,A^	21.93 ± 0.64 ^a,A^	8.25 ± 0.14 ^a,A^
	US-897	63.21 ± 4.58 ^a,A^	61.02 ± 1.07 ^a,A^	16.33 ± 0.38 ^ab,A^	9.26 ± 0.10 ^ab,A^
	US-942	70.48 ± 2.64 ^a,A^	62.03 ± 0.55 ^a,A^	9.07 ± 0.11 ^c,A^	4.55 ± 0.01 ^c,A^
Fresh	US-802	53.86 ± 2.29 ^abc,A^	65.32 ± 1.79 ^ab,A^	31.25 ± 1.64 ^a,B^	12.58 ± 0.60 ^a,B^
	US-897	45.23 ± 4.07 ^b,B^	70.17 ± 0.51 ^a,B^	25.82 ± 0.23 ^a.B^	9.49 ± 0.11 ^a,B^
	US-942	52.15 ± 3.53 ^c,B^	67.03 ± 1.85 ^b,B^	28.15 ± 1.33 ^a,B^	11.82 ± 0.40 ^a,B^

**Table 7:** Hesperidin, nobiletin, tangeretin, naringin, neohesperidin, and poncirin content profiles (ppm or ug/g dry weight) of fresh and steam-exploded (STEX) Hamlin, Valencia, US-897, US-942, and US-802. Paired, two-tailed *t*-test *p*-values are given in parenthesis.

Items	Hesperidin	Nobiletin	Tangeretin	Naringin	Neohesperidin	Poncirin
	ppm or ug/g Dry Weight					
Hamlin Fresh [22]	5625 ± 788	1068 ± 124	79 ± 16	nr	nr	nr
Hamlin STEX (170 °C 8 min) [22]	64,611 ± 3764	1137 ± 29	116 ± 19	nr	nr	nr
Valencia Fresh [22]	6372 ± 4901	994 ± 69	87 ± 17	nr	nr	nr
Valencia STEX (170 °C 8 min) [22]	39,712 ± 6836	786 ± 91	113 ± 18	nr	nr	nr
US-802 Fresh	0	0	0	15,014 ± 127	0	3974 ± 7
US-802 STEX	0	0	0	17,513 ± 212 (0.42)	0	3675 ± 149 (0.21)
US-897 Fresh	1273 ± 225	171 ± 4	118 ± 4	2905 ± 53	11,177 ± 1278	21,823 ± 174
US-897 STEX	1296 ± 135 (0.77)	177 ± 2 (0.11)	110 ± 1 (0.16)	2633 ± 145 (0.30)	10,911 ± 113 (0.83)	22,172 ± 216 (0.05)
US-942 Fresh	1427 ± 3	474 ± 12	417 ± 21	2757 ± 83	14,569 ± 21	5686 ± 12
US-942 STEX	1258 ± 22 (0.07)	355 ± 25 (0.05)	268 ± 4 (0.07)	2158 ± 352 (0.20)	11,026 ± 116 (0.02)	4072 ± 46 (0.02)

nr = not reported.

**Table 8:** Mass per hectare of components that can be recovered from US-802, US-897, and US-942 using steam explosion (STEX).

Items	Glucose	Peel Oil	Pectic Hydrocolloids	Flavonoid	Specific Flavonoid
	Dry Weight kg/Hectare				Compound Name
US-802	323	11	55	48	Naringin
US-897	158	27	24	29	Poncirin
US-942	304	58	59	27	Neohesperidin

## Data Availability

Data are contained within the article or Supplementary Material.

## Supplementary Materials

The following are available online at https://www.mdpi.com/article/10.3390/biology10121285/s1,Table S1: Glucose produced from steam explosion (stex); Table S2: Citrus Peel Based Hydrocolloid—Value Added Analysis*.
