# Supplementary material for: Steam Explosion (STEX) of Citrus × Poncirus Hybrids with Exceptional Tolerance to Candidatus Liberibacter Asiaticus (CLas) as Useful Sources of Volatiles and Other Commercial Products
Source: Biology (Basel). 2021 Dec 7;10(12):1285. doi: 10.3390/biology10121285 (PMC8698310; doi:10.3390/biology10121285)
Supplement: Supplementary file 1 [file biology-10-01285-s001.zip › biology-1447353_SI.pdf]

Table S2. Citrus Peel Based Hydrocolloid – Value Added Analysis*

A means of using a low valued material resulting from processing of citrus fruit for juice to produce a potentially high valued hydrocolloid based material in place of low valued citrus peel pellets ($0.08 – $0.10/lb).

Potential STEX Hydrocolloid Product Replacement Values

- Replace Hydroxy Propyl, Carboxy Methyl Guar @ $2.75/lb
- Replace LM/HM Pectin @ $6.00 – $12.00/lb
- Replace Alginate @ $5.00 – $10.00/lb Food Grade

Material Costs / One lb STEX Hydrocolloid Product

• Processed citrus peel		
1 lb @ $0.00/lb	=	$0.00
• Natural Gas		
0.03 Therm/lb @ $0.46/Therm	=	$0.02
• Steam		
0.22 lb/lb peel @ $0.05/lb	=	$0.01
**Total Material Cost / Pound Product**	**=**	**$0.03**

*Values taken from 2013-14
